# Supplementary material for: Throwbacks that move us: The dance-inducing power of nostalgic songs
Source: PLoS One. 2025 May 16;20(5):e0318766. doi: 10.1371/journal.pone.0318766 (PMC12083803; doi:10.1371/journal.pone.0318766)
Supplement: S1 Table — (PDF) [file pone.0318766.s001.pdf]

	Desire to tap	Desire to move	Desire to dance	Liking	Familiarity	Nostalgia
California Girls<br>Katy Perry ft. Snoop Dogg	74.09	69.21	62.73	69.87	95.27	74.95
OMG<br>Usher ft. will.i.am	69.46	66.73	57.62	64.07	85.51	60.41
Happy<br>Pharrell Williams	70.65	64.8	55.76	60.47	95.01	64.14
Bad Romance<br>Lady Gaga	75.41	70.49	62.97	75.63	94.83	74.33
Mirrors<br>Justin Timberlake	66.64	58.12	41.25	66.85	86.98	55.98
Uptown Funk<br>Mark Ronson ft. Bruno Mars	80.61	75.42	69.66	76.1	96	64.19
Thrift Shop<br>Macklemore & Ryan Lewis ft. Wanz	71.96	64.26	53.69	66.75	95.1	73.08
We Are Young<br>Fun. ft. Janelle Monáe	67.14	58.04	41.06	71.68	93.22	71.78
Forget You<br>CeeLo Green	76.39	73.46	63.25	76.29	93.95	72.57
Glad You Came<br>The Wanted	70.75	67.56	58.17	65.78	89.43	63.25
Party Rock Anthem<br>LMFAO ft. Lauren Bennett, GoonRock	71.81	69.81	66.88	63.17	94.67	76.44
Airplanes<br>B.o.B ft. Hayley Williams	69.16	59.84	40.85	72.09	93.99	75.76
Hey, Soul Sister<br>Train	66.75	56.36	39.23	68.89	93.97	74.31
Love the Way You Lie<br>Eminem ft. Rihanna	63.55	49.8	31.22	71.92	95.12	73.57
Rolling in the Deep<br>Adele	71.33	60.99	40.33	78.85	93.89	69.21
Tik Tok<br>Kesha	77.79	76.02	71.42	73.24	95.26	80.11
What Makes You Beautiful<br>One Direction	74.29	69.53	62.08	71.69	93.88	73.36
Firework<br>Katy Perry	66.98	61.09	55.05	63.1	96.34	69.37
Call Me Maybe<br>Carly Rae Jepsen	70.71	68.76	57.9	63.96	95.72	80.19
Dynamite<br>Taio Cruz	74.64	69.45	65.77	68.53	94.75	75.86
Maximum	80.61	76.02	71.42	78.85	96.34	80.19
Minimum	63.55	49.8	31.22	60.47	85.51	55.98
Mean (SD)	71.51 (4.27)	65.49 (6.85)	54.84 (11.75)	69.45 (5.15)	93.64 (2.91)	71.14 (6.48)
