# Supplementary material for: Throwbacks that move us: The dance-inducing power of nostalgic songs
Source: PLoS One. 2025 May 16;20(5):e0318766. doi: 10.1371/journal.pone.0318766 (PMC12083803; doi:10.1371/journal.pone.0318766)
Supplement: S2 Table — (PDF) [file pone.0318766.s002.pdf]

	Desire to tap	Desire to move	Desire to dance	Liking	Familiarity	Nostalgia
Circles<br>Post Malone	68.32	60.54	43.22	73.31	79.13	42.72
Rockstar<br>DaBaby fr. Roddy Ricch	61.87	56.25	43.60	56.81	70.65	31.46
Don’t Start Now<br>Dua Lipa	75.29	72.76	66.84	74.34	83.24	35.49
7 Rings<br>Ariana Grande	68.78	62.04	48.11	68.75	85.41	39.8
Adore You<br>Harry Styles	66.49	60.86	49.81	70.89	74.79	34.70
Bad Guy<br>Billie Eilish	68.90	58.89	44.02	67.07	86.79	35.13
Meant to Be<br>Bebe Rexha ft. Florida Georgia Line	52.75	43.14	26.42	48.57	76.71	31.7
God's Plan<br>Drake	70.46	64.90	54.21	70.55	84.21	51.98
Rockstar<br>Post Malone ft. 21 Savage	61.57	52.14	38.75	65.52	80.88	39.50
The Bones<br>Maren Morris	46.76	40.29	26.05	54.96	54.88	22.72
Girls Like You<br>Maroon 5 ft. Cardi B	58.14	50.07	37.88	62.02	83.95	36.60
I Like It<br>Cardi B, Bad Bunny & J Balvin	70.20	66.17	58.20	64.43	80.57	38.49
In My Feelings<br>Drake	64.07	59.25	49.49	61.95	79.04	49.17
Sucker<br>Jonas Brothers	66.28	61.57	51.55	68.31	82.05	38.90
Happier<br>Marshmello & Bastille	55.20	47.09	33.83	62.17	73.27	39.44
Memories<br>Maroon 5	45.25	35.71	21.84	56.59	76.30	40.00
Without Me<br>Halsey	60.15	50.14	31.44	65.45	79.60	40.29
The Middle<br>Zedd ft. Maren Morris and Grey	63.56	60.19	51.41	62.3	89.39	47.90
Blinding Lights<br>The Weeknd	77.36	72.65	67.07	77.55	90.85	43.45
Maximum	77.36	72.76	67.07	77.55	90.85	51.98
Minimum	45.25	35.71	21.84	48.57	54.88	22.72
Mean (SD)	63.23 (8.71)	56.56 (10.19)	44.41 (12.91)	64.82 (7.27)	79.56 (7.97)	39.92 (6.73)
