# Supplementary material for: Throwbacks that move us: The dance-inducing power of nostalgic songs
Source: PLoS One. 2025 May 16;20(5):e0318766. doi: 10.1371/journal.pone.0318766 (PMC12083803; doi:10.1371/journal.pone.0318766)
Supplement: S3 Table — Note. Participants were told that for items a–c, “Note that we are not asking you how much the music made you tap, move, or dance along, but how much you had a perceived feeling of wanting to tap, move, or dance to the music.”. (PDF) [file pone.0318766.s003.pdf]

**Term**	**Definition**
a. Tapping	This refers to how much the song makes you want to tap your finger/foot or bob your head to the music.
b. Moving	This refers to whether the song makes you want to move in any way. This includes both tapping along, dancing, or any other type of movement. This scale does not need to be a sum of the following two scales, but more of a general impression of how much it makes you feel like moving.
c. Dancing	This refers to how much the song makes you want to dance along. Note: in this instance dancing can be seen as any movement that is not simply tapping or bobbing along.
d. Enjoyment	This refers to how much you enjoy/like listening to the musical clip. This is different from wanting to move to the music, since you can like a piece of music without wanting to move to it or may want to move to a piece of music but not necessarily like it. Try to remember that these two concepts are distinct and that your score for one doesn't need to depend on the other.
e. Familiarity	This refers to whether or not you have heard this specific music piece before. This is not the same as if any of the music in the study reminds you of another song, but solely refers to whether or not you have heard the exact song before and how well you know it.
f. Nostalgia	Nostalgia is an emotion defined as ‘a sentimental longing or wistful affection for the past’. It can make you feel both good and bad, whether you mourn the past or recall the happiness you have felt in your life. In this study, you will be asked to rate how nostalgic the music makes you feel, which means how much the music makes you have feelings of sentimental longing for the past. The music could be from specific times of your life, associated with certain people, or music you listened to when you were younger. It may be bittersweet, positive or negative, or a mixture of the two.
